# Interim sample size re-estimation in the IIVOP trial

**DOI:** 10.1186/1745-6215-16-S2-P128

**Published:** 2015-11-16

**Authors:** Sarah Dawson, Mike Lee, Simon Bond

**Affiliations:** 1 Cambridge Clinical Trials Unit, University of Cambridge, Cambridge, UK; 2 Division of Anaesthesia, University of Cambridge, Cambridge, UK; 3 MRC Biostatistics Unit, Cambridge, UK

## 

The IIVOP trial is a cross-over trial which examines the effect of the drug ivabridine in reducing pain in an enriched healthy volunteer pain model. The trial was designed based on very limited information, and as such an interim analysis was built into the design to allow the re-estimation of the sample size requirements based on updated trial parameters.

The blinded data will be used to assess the assumptions regarding the variability of the primary endpoint. If appropriate and feasible the sample size may be increased to ensure that the study is not under-powered due to incorrect assumptions. In order to control for biases, the trial will not terminate early even if the interim sample size calculations suggests that enough patients have already been enrolled to reach the desired power.

The steps of the interim sample size re-calculation, including decisions made and results, will be presented.

